# Defensive chemicals of neighboring plants limit visits of herbivorous insects: Associational resistance within a plant population

**DOI:** 10.1002/ece3.4750

**Published:** 2018-12-11

**Authors:** Takashi Y. Ida, Kojiro Takanashi, Momoka Tamura, Rika Ozawa, Yoshitaka Nakashima, Takayuki Ohgushi

**Affiliations:** ^1^ Center for Ecological Research Kyoto University Otsu Japan; ^2^ Research Institute for Sustainable Humanosphere Kyoto University Uji Japan; ^3^ Faculty of Science Nara Women's University Nara Japan; ^4^ Present address: Faculty of Science Nara Women's University Nara Japan; ^5^ Present address: Institute of Mountain Science Shinshu University Matsumoto Japan

**Keywords:** associational effects, defensive traits, herbivore, neighborhood effect, neighboring plants, nicotine

## Abstract

Despite our understanding of chemical defenses and their consequences for plant performance and herbivores, we know little about whether defensive chemicals in plant tissues, such as alkaloids, and their spatial variation within a population play unappreciated and critical roles in plant‐herbivore interactions. Neighboring plants can decrease or increase attractiveness of a plant to herbivores, an example of a neighborhood effect. Chemical defensive traits may contribute to neighborhood effects in plant‐herbivore interactions. We examined the effects of nicotine in leaves (a non‐emitted defense chemical) on plant‐herbivore interactions in a spatial context, using two varieties of *Nicotiana tabacum *with different nicotine levels. A common garden experiment demonstrated that visits by grasshoppers decreased with increasing density of neighboring plants with a greater nicotine level. In contrast, visits of leaf caterpillars were not affected by neighbors, irrespective of nicotine levels. Thus, our results clearly highlighted that the neighborhood effect caused by the nicotine in leaves depended on the insect identity, and it was mediated by plant‐herbivore interactions, rather than plant‐plant interactions. This study demonstrates that understanding of effects of plant defensive traits on plant‐herbivore interactions requires careful consideration of the spatial distribution of plant defenses, and provides support for the importance of spatial context to accurately capture the ecological and evolutionary consequences of plant‐herbivore interactions.

## 1 INTRODUCTION

The effects of plant secondary metabolites, which play an important role in mediating interactions with herbivores, may be extended to the neighboring plants because those neighbors can defend plant tissues against herbivores visiting the focal plant interacting with the neighbors (e.g., reviewed by Bennett & Wallsgrove, 1994). Despite increased understanding of chemical defenses (Moore, Andrew, Külheim, & Foley, 2014) and their consequences for plant performance (Karban, 2011) and herbivore community composition (Kessler, 2004; Ohgushi, 2005), we know little about whether and how distribution of plants with secondary compounds in plant tissues influences preference and/or performance of herbivores. Neighboring plants can decrease or increase attractiveness of a focal plant to herbivores, an example of a neighborhood effect (Underwood, Inouye, & Hambäck, 2014). Two factors may contribute to neighborhood effect: density effects and associational effects. Density effects occur when the density of intraspecific neighbors affects the focal plant. When these effects occur, plants with a high density of neighbors are more or less attractive to herbivores and thus herbivores are more prevalent on high or low concentration of their host plants, respectively. In contrast, associational effects involve effects of interspecific neighboring plants on the focal plant. The focal plant receives benefits (e.g., discouraging herbivores on the focal plant) or costs (e.g., inducing herbivores to visit on the focal plant) from interspecific neighboring. Both density and associational effects can be applied to intraspecific interactions within a population (e.g., the effects of neighbor plants that are of the same or different phenotypes and/or genotypes, respectively). The spatial heterogeneity in a plant community or population influences abundance and foraging behavior of herbivores, thereby determining the damage level of plants (Champagne, Moore, Côté, & Tremblay, 2018; Stastny & Agrawal, 2014; Underwood et al., 2014). Whether the associational effect is beneficial (i.e., associational resistance) or detrimental (i.e., associational susceptibility) from the perspective of plants (Barbosa et al., 2009) is likely to be determined by the foraging behaviors of herbivores, depending on a fine‐scale assortment of plants with different palatabilities.

Most previous studies have considered only associational effects involved in interspecific interactions, such as plant species diversity (e.g., Andow, 1991). Similar arguments on associational effects would also apply to intraspecific interaction, that is, interactions among conspecific plants with different phenotypes and/or genotypes (Champagne et al., 2018; Coverdale, Goheen, Palmer, & Pringle, 2018). For example, within a population of *Solidago altissima*, genotypic diversity of co‐occurring plants decreased herbivory on the focal plants (i.e., associational resistance), although the mechanism was unclear (Genung, Crutsinger, Bailey, Schweitzer, & Sanders, 2012). Thus, a spatial perspective should be critical for better understanding the effects of plant defensive traits and plant‐herbivore interactions in a population context (Agrawal, Lau, & Hambäck, 2006; Ohgushi & Hambäck, 2015).

One possible mechanism underlying neighborhood effects is that neighboring plants with chemical defense in plant tissues may discourage herbivores from the local area where neighboring and focal plants coexist (repellent volatiles: Karban, 2007). Finch and Collier (2000) proposed an additional hypothesis, although it still remains untested. They assumed that herbivores visit host plants by detecting some cues, but if they happen to land on a non‐host neighbor (i.e., inappropriate landing) then they may move away immediately. If the herbivores recognize neighboring plants with unpleasant compounds (repellent chemicals in plant tissues and/or emitted volatiles, such as alkaloids) as an inappropriate landing, such host‐plant selection may cause positive associational effects on focal plants (i.e., neighbor‐contrast effect; Bergvall, Rautio, Kesti, Tuomi, & Leimar, 2006). Thus, herbivores' foraging behaviors would be modified by not only defensive traits of a focal plant but also those of neighbors, thereby creating neighborhood effects through the defensive trait. A growing body of studies address whether and how the presence of neighboring plants allows focal plants to gain benefits (i.e., associational resistance) or costs (i.e., associational susceptibility) (reviewed by Barbosa et al., 2009). Not surprisingly, herbivory acts as a selective pressure on defensive traits of plants (Futuyma & Agrawal, 2009). In addition, assessing the ecology and evolution of defensive traits requires understanding neighborhood effects because they could modify the selective pressures.

Many of the direct plant defenses involving secondary compounds are inducible, with activation after herbivore attack (Karban & Baldwin, 1997). Induced defenses are thought to be cost‐saving measures, where trade‐offs between the benefits of reduced herbivory and the costs of maintaining resistance lead to optimal resource allocation in plants (Coley, Bryant, & Chapin, 1985; Karban & Baldwin, 1997). Such trade‐offs could establish an evolutionary equilibrium of resource investment in defense depending on diverse local environments, resulting in variability of defensive traits within and among plant populations (Rausher, 1996). Given the costs and benefits of secondary compound production, associational resistance among genotypes and/or phenotypes within a species may allow plants to decrease investment in defense because neighboring plants provide the benefit of reduced herbivory. At a local population level, the increase in defense capacity due to associational resistance could discount per‐capita costs of resistance for individual plants. Intraspecific variation would contribute to the associational effects of plants interacting with herbivorous insects, although most previous studies have overlooked such intraspecific variations (but see Johnson, Lajeunesse, & Agrawal, 2006, Crawford and Rudgers 2013). Thus, we need to shed light on intraspecific variation and the frequency of plants with different defensive traits to understand the role of defensive traits in plant‐herbivore interactions.

In this study, we examined how alkaloid production in plants causes associational effects in plant‐herbivore interactions in an annual plant *Nicotiana tabacum* L., with a focus on fine‐scale spatial distribution of plants. *Nicotiana tabacum* synthesizes a toxic alkaloid, nicotine, in its belowground organs and rapidly translocates it to aboveground parts in response to herbivory. The inducible nicotine can protect the plants against generalist herbivores (Baldwin, 1988). To assess the neighborhood effects of alkaloids on plant‐herbivore interactions, we planted two *N. tabacum* varieties (i.e., genotypes) with different nicotine levels in a common garden, and examined effects of neighboring plants on herbivory of a focal plant. We hypothesized that nicotine of *N. tabacum* plants could contribute to positive neighborhood effects within a population. We evaluated neighborhood effects due both to density (i.e., effect of neighboring plants on the focal plant within same variety) and to association effects (i.e., effect of neighboring plants on the focal plant among varieties). Foraging decisions of herbivores may result in such associational effects for plants (Bergvall et al., 2006). For instance, herbivores' choice of a single plant from multiple plants at local scale may impose neighbor‐contrast effects (resistance or susceptibility), depending on the difference in palatability of neighbors (e.g., Hahn & Orrock, 2016). In addition, plant patch selection by herbivores should determine associational resistance or susceptibility of the patch composed of two or more genotypes with different defense capacities (e.g., Morrell & Kessler, 2017). In this study, we specifically addressed the following questions. First, do neighboring plants influence herbivory on the focal plant (i.e., neighborhood effects)? Second, do the direction and/or degree of the neighborhood effects differ depending on the nicotine levels of the neighbor plants? Third, do the neighborhood effects interact with defensive traits of the focal plants (i.e., counteracting or synergistic effects)?

## 2 MATERIALS AND METHODS

### 2.1 Study site and species

We studied *Nicotiana tabacum* L. in a common garden in the Center for Ecological Research, Kyoto University in Otsu, Japan (34^o^58'N, 135^o^57'E) from March to September 2015.* Nicotiana tabacum* is an annual plant that flowers from June to September in Japan, and is widely cultivated for use as tobacco. Flowering plants were about 1.2 m tall with terminal inflorescences producing bisexual flowers (median = 45, lower quartile = 22, upper quartile = 65) in our study site. In this study, we used high‐ and low‐nicotine varieties of *N. tabacum*: Burley 21 (hereafter, high‐nicotine variety (or plant)) and LA burley 21 (hereafter, low‐nicotine variety (or plant)) that are cultivars for commercial use (see Legg, Collins, & Litton, 1970). Morphological traits, such as size (see Supporting information Appendix S1) and shape, did not differ between the two varieties. Under herbivore‐exclusion, high‐nicotine plants had more than three times greater leaf‐nicotine concentrations (mean = 236.8 µg/g, lower *SE* = 213.6 µg/g, upper *SE* = 262.6 µg/g) than low‐nicotine plants (mean = 71.8 µg/g, lower *SE* = 64.7 µg/g, upper *SE* = 79.6 µg/g; Supporting information Table S1 of Appendix S2). Foliar damage by punching leaves induced nicotine production in leaves of high‐nicotine plants, but not in low‐nicotine plants, and the nicotine level in high‐nicotine plants increased with growing season. This upregulation of nicotine production occurred in newly produced leaves following leaf damage. Supporting information Appendix S2 provides details of methods for assessment of nicotine production. In addition, the volatile organic compounds (VOCs) emitted from leaves slightly differed between the varieties (see Supporting information Appendix S3). Specifically, high‐nicotine plants had 20% greater nicotine ((*S*)‐pyridine 3‐(1‐methyl‐2‐pyrrolidinyl)) than low‐nicotine ones, whereas low‐nicotine plants had 10% less *β*‐caryophyllene (terpenes, (*E*)‐*β*‐caryophyllene) than high‐nicotine plants. Thus, both nicotine concentration in leaves (hereafter, nicotine in leaves) and emission of nicotine from plants (hereafter, airborne nicotine) varied in the plants used in this study. In our preliminary experiments examining the effects of such differences in contents of nicotine on short‐term responses of herbivorous grasshoppers between varieties, we found the differences had little influence on their preference to high‐ and low‐nicotine plants at least in a short term (see Supporting information Appendix S4). Supporting information Appendix S4 provides more details about VOC measurements.

### 2.2 Experimental design

We grew seedlings of both *N. tabacum* varieties in flats in greenhouses during March 2015. Air temperature was regulated at 25/20°C (day/night), and all seedlings were watered daily. Seeds were provided by the Japan Tobacco Inc. After seedlings had four to six true leaves, they were individually transplanted into pots (12 cm in diameter) filled with commercial soil and fertilizer, and covered with fine‐meshed spectrally neutral vinylon cloth (Unitika vinylon #520, Unitika, Osaka), which slightly reduces light to 85%, to exclude insect attacks. All plants were watered as needed. Four hundred plants were randomly selected from the two varieties (high‐nicotine: 201, low‐nicotine: 199).

To evaluate the strength of neighborhood effects of *Nicotiana* plants and determine the spatial scale of the neighborhood effects, we directly assessed the neighborhood effects in plants assigned randomly within a plot (see Figure 2), rather than simple specific spatial design (e.g., the focal plants are exposed to different sets of spatial arrangements, such as densities and/or frequencies). Our experimental design can assess the distance‐dependent effects of neighborhood effects (see Results: Figure 2). We established a 12 m × 12 m plot in the common garden. Beginning on 15 May, 400 plants were randomly placed in the plot and all plants were mapped, so that we could account for spatial effects on plants (plant distribution). Water was supplied as needed until the end of the experiment. Beginning on 17 June, we recorded the number of insect visits on plants and open flowers, ending on 18 August and 10 September, respectively. We also measured stem diameter at ground level as an indicator of plant size on 1 June (day of year [doy] = 152, pre‐flowering stage), 21 July (doy = 202, flowering stage), and 9 August (doy = 221, fruiting stage).

### 2.3 Survey of insect visitors

To assess the plant‐insect interactions in relation to nicotine levels, we surveyed insects on high‐ and low‐nicotine plants at 2–3 days intervals throughout the observation period (in total 21 days, see Supporting information Appendix S5). On each observation day, we examined all plants and counted the number of insects (plot census). Insects observed on plants were classified into three groups: leaf caterpillars, grasshoppers, and seed predators (larvae of *Noctuidae* spp.). Grasshoppers and moth caterpillars consumed leaves, and moth adults oviposited on leaves (see Results for details). We divided observation time into three phases (i.e., pre‐flowering, flowering, and fruiting phases) based on plant reproductive stage and species composition of insects on plants. During pre‐flowering phase (doy 168–185, 7 observations during this phase), caterpillars (almost all were *Spodoptera litura*) and several species of grasshoppers (mostly *Atractomorpha lata*) were observed and they fed mainly on leaves (hereafter, leaf caterpillars and grasshoppers), whereas seed predators were only observed during fruiting phase (doy 208–230, 7 observations). In the intermediate flowering phase (doy 185–208, 7 observations), we observed only grasshoppers.

### 2.4 Data analysis

In all analyses, we fit generalized linear mixed models (GLMM: Stroup, 2013) as implemented with the GLIMMIX procedure of SAS version 9.4 (SAS Institute Inc., Cary, NC, USA). Mixed models were necessary for all analyses that accounted for spatial correlation in responses with a spatial‐power model, which proposes that the correlation in response among individual plants declines as a power function of their separation distance. The hypothesis testing for a covariance parameter was conducted based on the likelihood ratio test. The method of Kenward and Roger (1997) was used to adjust the (possibly fractional) denominator degrees of freedom for *F*‐tests to account for the estimated correlated responses.

For analyses of numbers of insects on plants, we summed data per plant within each observation phase. The analyses involved two steps. First, we assumed that effects between plants in close proximity (neighborhood effects: the influence of one plant on another that increases or decreases number of insects) exponentially decreased with distance between a given neighboring plant and the focal plant, and that the neighborhood effect is positively correlated with the size of the neighboring plant. Here, we postulated that close and large neighbors could influence the likelihood of herbivore visits, and that this effect weakens as the distance to the focal plant increases, as neighborhood effects are limited to a relatively close area. We adopted the exponential decay model (Devaux, Lavigne, Austerlitz, & Klein, 2007) to describe the effects of neighboring plants on the focal individual plant. The simplest kernel function (i.e., a probability density function) was characterized by one scale parameter *a* as following:

(1) $$\gamma \left(a,r_{i,j}\right)=\frac{1}{2\pi a^{2}}e^{\left(-\frac{r_{i,j}}{a}\right)},$$

where *r_i,j_* is the distance between a given neighboring plant *j* and the focal plant *i*. These functions correspond to the limit between the thin‐tailed and fat‐tailed effect function. Such weighting that depends on distance to the focal plants allows us to estimate the magnitude of decline of neighborhood effects (e.g., slope of the decline). Next, we assumed that the neighborhood effect of a given neighboring plant *j* on the focal plant (*N_i,j_*) might depend on plant size as following:

(2) $$N_{i,j}=\gamma \left(a,r_{i,j}\right)\times S_{j},$$

(3) $$N_{i}=\Sigma \gamma \left(a,r_{i,j}\right)\times S_{j},$$

where *S_j_* is plant size expressed by stem diameter of the plant *j*. Thus, neighborhood effect of a single plant (*N_i,j_*) on the focal plant *i* was weighted by the size of that plant *j*. The focal plant *i* received the sum of the neighborhood effects (*N_i_*) of all high‐ or low‐nicotine plants in the plot, respectively, and this was defined as the neighborhood effects of high‐ or low‐nicotine plants (see Results). Thus, neighborhood effects can be expressed as the sum of individual neighborhood effects of neighboring plants, which depend on identity, frequency, and distances to the focal plant of the neighbors. We obtained neighborhood effects of the neighboring plants on a focal plant in two ways: effects of high‐nicotine plants and those of low‐nicotine plants in the plot. This was because we assumed that the neighborhood effect varied between varieties of sources. We examined the effects of focal plant variety (high‐ or low‐nicotine varieties), focal plant size (represented as ln[stem diameter]), neighborhood effects of high‐nicotine plants, and neighborhood effects of low‐nicotine plants on the number of insects. We used a GLMM with a Poisson distribution (ln‐link function) for analyses of leaf caterpillars and grasshoppers and a binomial distribution (logit‐link function) for analysis of seed predators. Possible interaction terms between the focal plant variety and other covariate variables (i.e., focal plant size and neighborhood effects of neighboring plants) were included in the models to assess the interacting effects. Non‐significant interactions were eliminated from the final model by backward elimination (*α* = 0.05). Before the analysis, number of seed predators on plants was translated into binary data (presence or absence) because we rarely found multiple visits per observation (12 plants out of 322 fruiting plants received ≥2 insect visits). To obtain the proper scale parameter *a*, we repeated the GLMMs with variable *a* (1,000 candidates from 0.1 to 10 for each) to select the best‐fit model. Only in the case of the analysis of seed predators, we additionally considered the neighborhood effects of fruiting high‐nicotine plants, neighborhood effects of fruiting low‐nicotine plants, and fruit number of the focal plant as an alternative to stem diameter, because seed predators consume fruits so that fruits of the focal plant and/or neighboring plants should be the relevant attractants. This analysis was conducted for fruiting plants.

To facilitate interpretation, we present results for a particular factor or covariate adjusted for the effects of other components in the statistical models. For categorical factors, we present least‐squares means and their standard errors (Milliken & Johnson, 1984). We back‐transformed results from the scale of the link function to the original scale of measurement, which results in asymmetrical standard errors.

## 3 RESULTS

### 3.1 Insect visits to plants

In the pre‐flowering phase, we observed 752 insects on 400 plants. Most of them (81%) were leaf caterpillars and the remaining were grasshoppers. The number of leaf caterpillars on all plants peaked on day of year (doy) 173, whereas that of grasshoppers increased as time throughout the observation (see Supporting information Appendix S5). Distributions of leaf caterpillars and grasshoppers during the pre‐flowering phase were highly correlated with spatial distribution of plants within a plot ($\chi_{2}^{2}$ > 31.5, *p* < 0.001), but not by focal plant traits (i.e., variety and stem diameter) or characteristics of neighboring plants (i.e., neighborhood effects of high‐ and low‐nicotine plants) (Table 1).

**Table 1:** Results of generalized linear mixed models of the effects of variety of focal plants (high‐ or low‐nicotine variety), stem diameter, neighboring high‐ and low‐nicotine plants (neighborhood effects [NE] of high‐ and low‐nicotine plants, respectively, to a given plant), fruit number, and neighboring fruiting high‐ and low‐nicotine plants (neighborhood effects [NE] of high‐ and low‐nicotine fruiting plants, respectively, to a given plant) on numbers of leaf caterpillars, grasshoppers, and seed predators on *Nicotiana tabacum*. Analyses of seed predators were conducted twice with ln(stem diameter) or ln(fruits) because these were strongly correlated. Scale parameters were obtained by comparison of 1,000 trials (see main text for more detail)

Factor	Pre‐flowering phase		Flowering phase	Fruiting phase	
	Leaf caterpillar	Grasshopper	Grasshopper	Seed predator	Seed predator
Scale parameter	4.24	4.37	0.41	0.7^a^, 0.4^b^	0.7^a^, 0.4^b^
Variety	*F*_1,372.0_ = 0.05	*F*_1,395.0_ < 0.01	*F*_1,390.0_ = 13.07***	*F*_1,313.0_ = 0.61	*F*_1,313.0_ = 0.14
Ln(stem diameter)	*F*_1,395.0_ = 1.36	*F*_1,395.0_ = 0.03	*F*_1,390.0_ = 20.17***	*F*_1,313.0_ = 10.72**	…
NE of high‐nicotine plants	*F*_1,353.8_ = 1.37	*F*_1,25.56_ = 3.87	*F*_1,325.5_ = 15.02***	*F*_1,74.92_ = 2.44	*F*_1,49.27_ = 1.09
NE of low‐nicotine plants	*F*_1,371.7_ = 0.53	*F*_1,44.01_ = 0.51	*F*_1,303.9_ = 0.06	*F*_1,313.0_ = 2.37	*F*_1,313.0_ = 1.56
Ln(fruits)	…	…	…	…	*F*_1,313.0_ = 8.55**
NE of high‐nicotine fruiting plants	…	…	…	*F*_1,313.0_ = 0.18	*F*_1,313.0_ = 0.11
NE of low‐nicotine fruiting plants	…	…	…	*F*_1,313.0_ = 3.75	*F*_1,313.0_ = 2.65
Spatial correlation	$\chi_{2}^{2}$ = 308.38***	$\chi_{2}^{2}$ = 31.55***	$\chi_{2}^{2}$ = 27.91***	$\chi_{2}^{2}$ < 0.01	$\chi_{2}^{2}$ = 0.08

a Scale parameter for neighborhood effects of high‐nicotine and low‐nicotine plants in the plot.

b Scale parameter for neighborhood effects of high‐nicotine and low‐nicotine fruiting plants in the plot.

** *p* < 0.01

*** *p* < 0.001.

**Figure 1:**
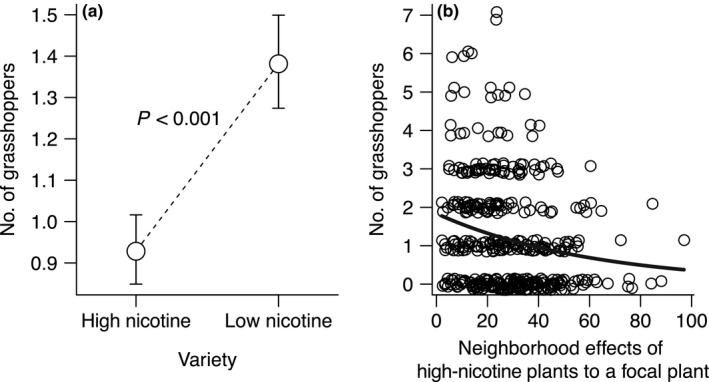
(a) Differences in the least‐squares mean (±*SE*) grasshoppers observed on plants between varieties with high‐nicotine and low‐nicotine levels in *Nicotiana tabacum*. (b) Overall neighborhood effects of all high‐nicotine plants within the plot on either high‐nicotine or low‐nicotine plants measured by the number of grasshoppers in flowering phase in *N. tabacum*. Neighborhood effects were obtained by summing the neighborhood effects of all neighboring plants of each variety considering the distance to the focal plant and size of the neighbor (see the subsection of Data analysis for more detail). The regression line shown was back‐transformed from GLMM

**Figure 2:**
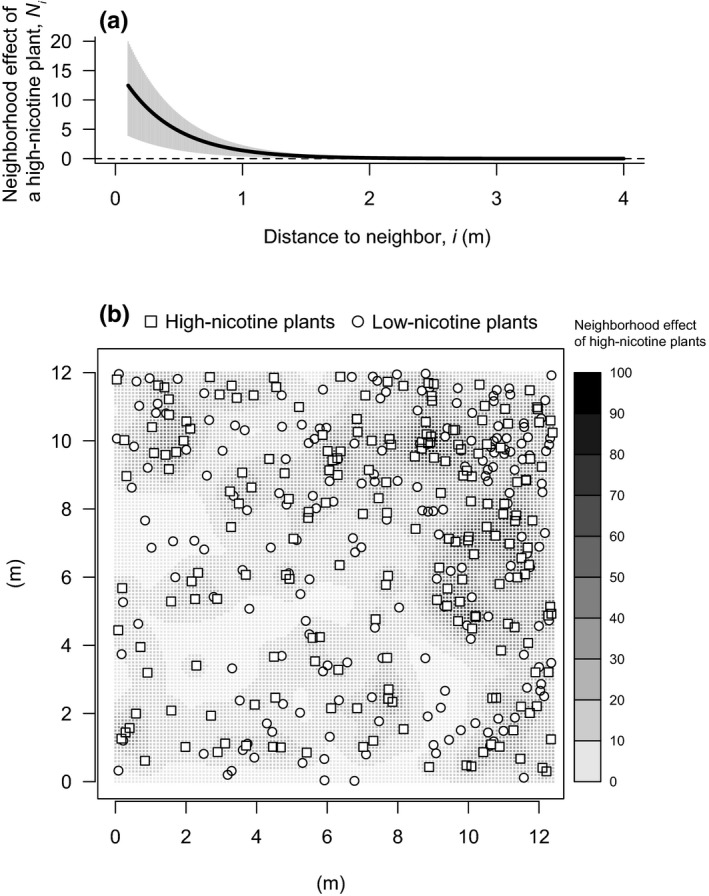
(a) Neighborhood effects of a single, average‐sized (in terms of stem diameter) high‐nicotine plant on grasshoppers present on plants in the flowering phase. Relation of associational effect of a neighboring high‐nicotine plant on grasshopper visits to a focal plant in the flowering phase to distance between them (*N_i_*). (b) Spatial distributions of plants in the study plot and neighborhood effects of high‐nicotine plants obtained by the best‐fit model with 0.41 as parameter *a*. Darker shading indicates greater neighborhood effects of high‐nicotine plants (i.e., greater positive effects of neighborhood effect). See text for details about how neighborhood effects were calculated

In the flowering phase, 509 grasshoppers were observed, but leaf caterpillars were no longer observed. The grasshoppers' visits were spatially correlated among plants. After accounting for the effects of spatial correlation, grasshopper visits differed between the varieties (49% more in low‐nicotine plants than high‐nicotine plants, Figure 1a) and proportionally increased with stem diameter (i.e., plant size, partial regression coefficient: *b* ± *SE* = 1.130 ± 0.252; comparison with *b* = 1 expected with a proportional increase, *t*_390_ = 0.52, *p* = 0.30, Table 1). Furthermore, neighboring high‐nicotine plants, but not low‐nicotine plants, influenced grasshoppers on the focal plant (neighborhood effects of high‐nicotine and low‐nicotine plants in the plot on a given plant: Figure 1b, Table 1). Grasshoppers decreased with neighborhood effects of high‐nicotine plants (*b* ± *SE* = −0.016 ± 0.004: Figure 1b), whereas they were not influenced by neighbors with low‐nicotine contents (Table 1). In the best‐fit model, scale parameter *a* for the model of grasshopper visits was 0.41. Thus, the influence of a given neighboring plant *i* on grasshopper visits to the focal plant exponentially decreased with increasing distance between plant *i* and the focal plant. For instance, the neighborhood effect of a plant at 0.1 m distance was 12.46, and declined by 95% and 99% at 1.4 m and 2.0 m, respectively. Thus, it tended toward almost zero at approximately 1.4–2.0 m distance when plant *i* had an average stem diameter (Figure 2a). For instance, neighborhood effect of a single high‐nicotine plant that has an averaged size of stem diameter and is 30 cm apart from the focal plant is 7.65 (Figure 2a). In this situation, grasshoppers on the focal plant decreased in number by 11%, compared to a plant without neighboring plants (Figure 1b). To facilitate interpretation, we back‐transformed results of GLMM for grasshoppers during the flowering phase to express the effects in space (at each fine mesh [10 cm × 10 cm]). High‐nicotine plants had neighborhood effects that tended to be greater for focal plants surrounded by more high‐nicotine plants (Figure 2b).

In the fruiting phase, larger plants with more fruits received more seed predators, regardless of variety or neighboring plant traits and distributions (Table 1). The probability of seed predators increased with stem diameter of the focal plant (*b* ± *SE* = 3.290 ± 1.005) and with fruit number (*b* ± *SE* = 0.917 ± 0.314).

## 4 DISCUSSION

### 4.1 Neighborhood effects for *Nicotiana tabacum*

Our results clearly demonstrated that neighborhood effects caused by neighbors with high nicotine levels reduced herbivorous insects attacking focal plants. We found that neighboring high‐nicotine plants discouraged grasshoppers that visit *Nicotiana* plants (both high‐ and low‐nicotine varieties) in the flowering phase. This response occurred in both high‐ and low‐nicotine varieties with high‐nicotine neighbors, indicating that the neighborhood effect of plants with greater nicotine levels contributes to the reduction in herbivore visits, and that both a density effect and an associational effect contributed to the neighborhood effect. This pattern contrasts with our laboratory experiments (Supporting information Appendix S3 and S4), where neither airborne nicotine nor nicotine in leaves affected grasshopper preference for *Nicotiana* plants, at least during the experimental period. Furthermore, neighborhood effects were not detected during the pre‐flowering phase in the field. Thus, neighborhood effects via nicotine did not arise soon after an initiation of the plant‐herbivore interaction, suggesting that the sufficient time to feed on high‐nicotine plants is required for grasshopper learning.

To our best knowledge, previous studies focused on the presence of neighbors that might influence focal plant performance and what exact plant traits generate neighborhood effects remained unclear in most cases. A typical neighborhood effect in defensive traits against herbivorous insects is plant communication using volatile organic compounds (eavesdropping: Kost & Heil, 2006, Karban, Shiojiri, Huntzinger, & McCall, 2006). In this case, the neighborhood effect is due to airborne volatiles, which are effective within relatively short distance (e.g., <10–15 cm for interspecific interaction between sagebrush and tobacco (Karban, Baldwin, Baxter, Laue, & Felton, 2000; Karban, Maron, Felton, Ervin, & Eichenseer, 2003), <60 cm for intraspecific interaction of sagebrush (Karban et al., 2006)). In contrast, we could detect greater neighborhood effects: an average‐sized plant was affected by a neighbor growing 1.4–2.0 m away. This result suggests that the neighborhood effect found in this study was unlikely to be caused by airborne volatile substances alone. Thus, effects of nicotine in leaves as well as airborne nicotine extend beyond the individual plant to affect associated neighbors (extended phenotype: Dawkins, 1982, Whitham et al., 2003, Johnson et al., 2006). Not surprisingly, it has been well accepted that the production of defensive chemicals by plants (such as nicotine) has ecological and evolutionary consequences based on trade‐offs between resource investments in defense traits and other sinks, such as growth and reproduction (Halitschke, Hamilton, & Kessler, 2011; Kessler, 2004; Kessler, Halitschke, & Poveda, 2011; McKey, 1974; Walling, 2000). This study clarified the importance of understanding the scale and magnitude of neighborhood effects caused via plant defensive traits in a population/community context.

### 4.2 Consequence of nicotine in leaves for neighborhood effects

The mechanisms creating neighborhood effects in *N. tabacum* involve not only nicotine levels of plant genotypes but also the foraging behavior of grasshoppers. Although the neighborhood effects of high‐nicotine plants were spatially limited (approximately <1.4–2.0 m distance from a focal plant in our study system, Figure 2a), this distance was much greater than found in previous studies of associational resistance related to specific defensive traits of plants interacting with herbivorous insects having similar mobilities with grasshoppers (Karban et al., 2000, 2003, 2006). This discrepancy in the effective distance is likely due to the proximate factors causing neighborhood effects. The previous studies of associational resistance using secondary compounds of plants including *Nicotiana* species (Karban et al., 2000, 2003; Kost & Heil, 2006; Tscharntke, Thiessen, Dolch, & Boland, 2001) have focused on plant‐plant communication via volatiles (i.e., eavesdropping). In this context, herbivore‐induced volatiles released by damaged neighboring plants make the focal plants more resistant by upregulation of defense level before herbivore arrival or by priming of defenses. Such airborne signaling can be strongly influenced by local abiotic conditions, such as wind direction and speed (Heil & Karban, 2009), hence these factors may limit the effective distance between volatile emitters and receivers.

This study suggests another possible mechanism responsible for the neighborhood effect involving nicotine in leaves at the fine patch scale. Specifically, long‐distance foraging movements of herbivores after visits to neighboring high‐nicotine plants may create neighborhood effects for the focal plant (Hambäck, Inouye, Andersson, & Underwood, 2014; Potting, Perry, & Powell, 2005), although further studies are required to determine how far a single grasshopper moves between plants after encountering each genotype. It should be noted that the repellent outcome using defensive chemicals in plant tissues (i.e., nicotine in leaves) via plant‐herbivore interactions could act over a wider range than airborne signaling with emitted volatiles via plant‐plant communication (e.g., Karban et al., 2006). Whether the outcome is positive or negative neighborhood effects for a focal plant largely depends on herbivore behavior after feeding on neighboring plants; for example, highly‐defended neighboring plants may discourage herbivores from remaining in the area (Root, 1973). Species‐specific responses to neighborhood effects among herbivorous insects may occur due to differences in their mobility and thus their ability to seek more palatable plants. For example, the lepidopteran caterpillars grew on the plant where their mothers oviposited in our study (*personal observation*). Thus, sedentary caterpillars might not choose host plants themselves, resulting in a weakened neighborhood effect. In contrast, long feeding times and fidelity to particular plant species (Chambers, Sword, Angel, Behmer, & Bernays, 1996) may allow grasshoppers to learn and to select plants based on food quality (Bernays, Bright, Gonzalez, & Angel, 1994; Chambers et al., 1996), although an innate response is another possibility for some insects in natural conditions (De Roode, Lefevre, & Hunter, 2013). Such learned foraging behavior in grasshoppers, including avoidance of plants with toxic secondary compounds (Freeland & Janzen, 1974) and/or maintenance of appropriate nutrient balance (Rapport, 1980; Westoby, 1978), may translate into improved growth performance (Bernays et al., 1994; Dukas & Bernays, 2000). The more toxic plant secondary compounds that grasshoppers encountered, for example, the more likely they were to leave the patch (patch departure rule, Charnov 1976). In this way, food selection by grasshoppers may promote emigration from patches with high‐nicotine genotypes, resulting in enhanced neighborhood effects via reduction of grasshoppers' colonization (i.e., positive neighborhood effect) for co‐occurring plants during the flowering phase. Thus, we conclude that the neighborhood effect mediated by nicotine in leaves was caused by plant‐herbivore interactions, rather than plant‐plant interactions.

The advantage of neighborhood effect via defensive chemicals in plant tissues (relative to airborne volatiles) has further implications for plant performance. Eavesdropping on the emissions of volatiles from neighboring high‐nicotine plants should be a good way to know in advance when the plants should invest more resources to defense. While such plant communication is beneficial for avoidance of herbivory (Kost & Heil, 2006; Tscharntke et al., 2001), plant fitness may be reduced by greater investment in defense. For instance, Karban and Maron (2002) reported that, in some years, the likelihood of frost damage increased for plants that induced resistance in response to plant communication (i.e., eavesdropping) from artificially clipped neighbors. In contrast, the neighborhood effect mediated by non‐volatiles does not require such additional costs for induced defense in receiver plants. This cost‐saving strategy can enhance the plant's vegetative and reproductive performance.

### 4.3 Contrasting defense strategies within a plant population

Our results indicate that irrespective of nicotine levels, the focal plants (i.e., recipients) can gain the benefits of positive effects by high‐nicotine neighbors, which are donors in the facilitation (i.e., asymmetric donor‐recipient relationship). Evolutionary theories (Leimar & Tuomi, 1998; Tuomi, Augner, & Nilsson, 1994) exploring the significance of associational effects (i.e., effects of neighbors with a different type, defensive trait for example, on the focal plant) on natural selection for herbivore resistance predict that neighborhood effects arise through a frequency dependent process in a patch scale. Moreover, associational effects may arise from interactions between herbivores and multiple plant phenotypes within a patch (Tuomi & Augner, 1993; Tuomi et al., 1994) because neighboring species would affect herbivore density in the patch (e.g., Agrawal, 2004). Thus, the spatial distribution of plants should contribute to population‐level processes of herbivore attack.

In this context, positive neighborhood effects could allow plants to adopt two contrasting strategies against herbivores: *fighter* and *sneaker*. The fighter genotype invests greater resources in chemical defense against herbivores. Such a genotype would experience benefits from reduced herbivory but pay a greater cost of resistance. For instance, Baldwin (1998) demonstrated that induced nicotine production in *Nicotiana attenuata* led to a significant fitness loss if plants were artificially protected from herbivores using fencing and insecticide spray. Hence, a fighter genotype could gain low (Baldwin, 1999) but presumably relatively constant performance in growth and reproduction because nicotine production is beneficial even after deducting the costs of production (Baldwin, 1998). On the other hand, the sneaker genotype invests little in chemical defense under the patronage of neighboring fighter genotypes and will gain more reproductive success if the plant can successfully escape from herbivory. This results in potentially high growth and reproductive performance of this genotype but likely suppression by severe herbivory. These strategies can lead to coexistence of genotypes with both lower and higher investment in defense in the local patch. Our experimental study strongly emphasizes that the effectiveness of defensive traits of a given plant is context dependent. In particular, the spatial arrangement (i.e., identity, frequency, and distance to the focal plant of neighbors) of two genotypes with different nicotine levels within a population matter. Thus, defensive traits of individuals may scale up to a population level, making it important to account for how contrasting genotypes affect population dynamics. Furthermore, recent studies demonstrated that intraspecific plant genetic diversity can act as an important factor in shaping herbivore communities on plants (Cook‐Patton, McArt, Parachnowitsch, Thaler, & Agrawal, 2011, Genung et al., 2012, Crawford and Rudgers 2013). Exploring the precise plant‐herbivore interactions in nature thus requires further attention to the scope of neighborhood effects mediated by defensive traits and their genotypic variations in a spatial context.

## 5 CONCLUSION

Our study clearly demonstrated that understanding of the effects of defensive traits in plants as a consequence of plant‐herbivore interactions requires an explicit consideration of the spatial distribution of plants. It is essential to recognize that plant populations are spatially structured by multiple and diverse genotypes and/or phenotypes. We emphasize this spatial perspective for understanding trait‐mediated indirect effects in plant‐herbivore interactions (see Ohgushi & Hambäck, 2015) and presumably their evolution because scaling‐up these spatial effects may contribute to ecological and evolutionary dynamics (Strauss, 1991; Underwood et al., 2014).

## CONFLICT OF INTEREST

None Declared.

## AUTHORS CONTRIBUTION

TYI and TO involved in conceptualizing and planning all experiments. KT involved in nicotine analysis. TYI and MT involved in field experiment. RO involved in VOC measurements; YN involved in grasshoppers' choice experiment. All authors involved in writing and editing the manuscript.

## DATA ACCESSIBILITY

All data are available at the Dryad Digital Repository: http://doi:10.5061/dryad.6mn56r9.
