# Supplementary material for: Defensive chemicals of neighboring plants limit visits of herbivorous insects: Associational resistance within a plant population
Source: Ecol Evol. 2018 Dec 11;8(24):12981–90. doi: 10.1002/ece3.4750 (PMC6309010; doi:10.1002/ece3.4750)
Supplement: Supplementary file 1 [file ECE3-8-12981-s001.docx]

## Electronic supplementary information

**Defensive chemicals of neighboring plants limit visits of herbivorous insects: associational resistance within a plant population**

**Takashi Y. Ida** ^*,^ †^, 1^, **Kojiro Takanashi** ††, ^2^, **Momoka Tamura** ^3^, **Rika Ozawa** ^1^, **Yoshitaka Nakashima** ^1^ **and Takayuki Ohgushi** ^1^

^1^Center for Ecological Research, Kyoto University, Otsu, 520-2113, Japan; ^2^Research Institute for Sustainable Humanosphere, Kyoto University, Uji, 611-0011, Japan and ^3^Faculty of Science, Nara Women's University, Nara, 630-8506, Japan

^*^ Corresponding author. E-mail: tyida@cc.nara-wu.ac.jp, tel: +81-(0)742-20-3054

† Current address: Faculty of Science, Nara Women’s University, Nara, 630-8506, Japan

†† Current address: Institute of Mountain Science, Shinshu University, Matsumoto, 390-8621, Japan

Appendix S1. Morphological traits

Appendix S2. Nicotine production

Appendix S3. Volatile organic compounds

Appendix S4. Grasshoppers' choice of plants

Appendix S5. Seasonal variation in flower production and insect visit**Appendix S1. Morphological traits**

We compared morphological traits during early growing season in 2015 between high-nicotine and low-nicotine varieties. We measured stem diameter on 1 June (pre-flowering stage) and 21 July (flowering stage), and counted leaf number on 10 June (pre-flowering stage) and 22 June (flowering stage) on the 400 plants. Plants were cultivated in a common garden in the Center for Ecological Research as part of our main experiment (see main text). During pre-flowering, herbivore attacks were extremely rare (see Appendix S5), so that before anthesis we could assess the potential size of each variety under little herbivory.

We analyzed both stem diameter and leaf number by fitting generalized linear mixed models (GLMM: Stroup 2013) with the GLIMMIX procedure of SAS version 9.4 (SAS Institute Inc. 2013). Mixed models were necessary for all analyses; we accounted for spatial correlation in responses with a spatial-power model, which proposes that the correlation in response among individual plants declines as a power function of their separation distance. The hypothesis testing for a covariance parameter was conducted based on the likelihood ratio test. The method of Kenward and Roger (1997) was used to adjust the (possibly fractional) denominator degrees of freedom for *F*-tests to account for the estimated correlated responses. We examined the effect of variety (i.e., high-nicotine vs. low-nicotine) on the stem diameter or leaf number at each observation day, using GLMM that involved a gamma or Poisson distribution (both: ln-link function), respectively.

Stem diameter and leaf number did not statistically differ between the varieties (stem diameter: *F*_1,371.1_ = 1.50, *P* = 0.22 [pre-flowering] and *F*_1,65.4_ = 2.22, *P* = 0.14 [pre-flowering], leaf number: *F*_1,398_ = 0.70, *P* = 0.40 [pre-flowering] and *F*_1,398_ < 0.01, *P* = 0.99 [flowering]). Thus, morphological traits at least from the perspective of plant size did not differ between varieties that were used in main experiments.

# **Appendix S2. Nicotine production**

In tobacco, herbivory induces production of several defensive chemicals including nicotine (Lou and Baldwin 2003). To examine the herbivory-induced nicotine production and its variation between Burley 21 (hereafter, high-nicotine plants) and LA burley 21 (hereafter, low-nicotine plants), we compared induced plant resistance to foliar damage between varieties. *Nicotiana tabacum* seedlings were cultivated in a greenhouse under 25/20 ^o^C (day/night) and herbivores were excluded (20 Aug, 2015 - 25 Nov, 2015). All plants were watered daily. We conducted following two experiments.

The first experiment examined whether leaf damage of neighboring leaves induced nicotine production in other leaves within a same plant. We punched 20-30 holes (1 cm in a diameter) on a given leaf at a middle positioned stalk to imitate foliar herbivory. Then, we sampled leaf disks (1 cm in a diameter) from a neighboring leaf of the artificially damaged leaf on 1, 2, 3, 5, 7, 9, 12, and 15 days after leaf damage. We conducted a time-course experiment with different plants for each time (i.e., not repeated measures from single plant) because leaf sampling may influence foliar damage and plant resistance. In total, 122 one-month old non-flowering plants were randomly selected and assigned to greenhouse benches just before the experiment. We randomly divided 96 plants (high-nicotine: 48, low-nicotine: 48) subject to leaf damage treatment into eight groups of 6 individual each (1, 2, 3, 5, 7, 9, 12, and 15 days after leaf damage). Two low-nicotine plants were eliminated from analyses because they were accidentally damaged. One low-nicotine plant in the 3-days-after-damage group was accidentally damaged and excluded from all analyses. We also divided the remaining 26 plants (high-nicotine: 13, low-nicotine: 13) subject to no leaf-damage as a control into three groups of 4-5 individual each (0 day, i.e., timing of leaf damage and 7 and 15 days after leaf damage).

The second experiment was conducted to examine whether leaf damage induced nicotine production in leaves produced after the damage. On 11 November (46 days after the experimental onset), we resampled leaves from plants that were manipulated in the first experiment and were at the flowering stage. These leaves were produced after damage. From plants used in the first experiment, 17 plants (high-nicotine: 9, low-nicotine: 8) were randomly selected. We also harvested leaves from 18 unmanipulated plants (i.e., plants were not damaged, high-nicotine: 9, low-nicotine: 9) as control.

All leaf-disks sampled were stored at-30 ^o^C until High Performance Liquid Chromatography (HPLC) analysis. Three to six leaf disks (ca. 100 mg in total) per leaf were ground using a Multi-Beads Shocker (model MB601NIHS, Yasui Kikai Co. Osaka, Japan), and extracted with 3 volumes (v/w) of ice-cold extraction solvent (50% MeOH, 50% EtOH) for 30 min at 4 ^o^C. After centrifugation at 8000g for 10 min, the supernatant was filtered using Minisart RC4 (0.45 µm, Sartorius, Goettingen, Germany). The filtrate was injected into a HPLC system (A Shimadzu LC-10AD) with a Cosmosil 5C_18_-MS-II column (4.6 mm x 250 mm, Nacalai Tesque, Kyoto, Japan) at 30 ^o^C. The mobile phase was MeOH/10 mM NaHPO_4_ (pH7.0) = 40/60 at a flow rate 0.7 mL/min, and eluent was monitored at 260 nm.

We analyzed the effects of variety, leaf damage, and time sequence from leaf damage on nicotine production with generalized linear models (GLM) with gamma distribution (ln-link function) using SAS 9.4 (SAS Institute Inc.). For the first experiment, the analysis included variety (high-nicotine or low-nicotine) and damage treatment (damaged or undamaged) as fixed factors and time since leaf damage as a covariate. For the second experiment, the analysis included variety (high nicotine or low nicotine) and damage treatment (damaged or undamaged) as fixed factors. Possible interactions were considered; non-significant interactions were eliminated from the final model by backward elimination (α = 0.05).

Leaf damage strongly increased the nicotine production in the adjacent leaves that had not received leaf damage and newly produced leaves of high-nicotine plants, but not in low-nicotine plants. In the first experiment, effects of leaf damage on nicotine production differed between varieties, and nicotine production was induced by leaf damage in high-nicotine plants, but not in low-nicotine plants (Fig. S1a). Also, nicotine content increased with time in high-nicotine plants (partial regression coefficient, *b* ± SE = 0.049 ± 0.009; comparison with *b* =0, *t*_115_ = 5.29, *P* < 0.0001), but not in low-nicotine plants (*b* ± SE = -0.010 ± 0.009; comparison with *b* =0, *t*_115_ = -1.03, *P* = 0.30: Fig. S1b). Thus, nicotine production was induced in high-nicotine plants by leaf damage with the difference in nicotine content between varieties increasing over time. The second experiment showed that leaf damage affected nicotine production in the newly produced leaves following leaf damage. In addition, the nicotine content between damaged and undamaged plants differed in high-nicotine plants, but not in low-nicotine plants (Fig. S1c, Table S1b). Thus, upregulated nicotine production in high-nicotine plants induced by leaf damage (i.e., first experiment) was maintained in newly produced leaves (i.e., second experiment).

**References**

Lou Y, Baldwin IT (2003) *Manduca sexta* recognition and resistance among allopolyploid *Nicotiana* host plants. Proc Natl Acad Sci USA 100: 14581-14586. doi:10.1073/pnas.2135348100

| Table S1. Results of generalized linear models of the effects of variety with high-nicotine or low-nicotine level and foliar damage (first experiment), and variety and damage experience (second experiment) on the nicotine level in adjacent leaves and newly-emerged leaves of *Nicotiana tabacum*, respectively. | |
| --- | --- |
| Factor | Nicotine level  (µg/g in leaf) |
| First experiment  Variety (high-nicotine or low-nicotine) |  |
|  | *F*_1,115_ = 74.50*** |
| Leaf damage (damaged or undamaged) | *F*_1,115_ = 1.71 |
| Variety x Leaf damage | *F*_1,115_ = 8.09* |
| Days from experiment onset | *F*_1,115_ = 9.19** |
| Variety x Days from experiment onset | *F*_1,115_ = 20.12*** |
| Second experiment |  |
| Variety (high-nicotine or low-nicotine) | *F*_1,31_ = 356.49*** |
| Damage experience (damaged or undamaged) | *F*_1,31_ = 3.54 |
| Variety x Damage experience | *F*_1,31_ = 7.00* |
| *** *P* < 0.001, ** *P* < 0.01, * *P* < 0.05. |  |


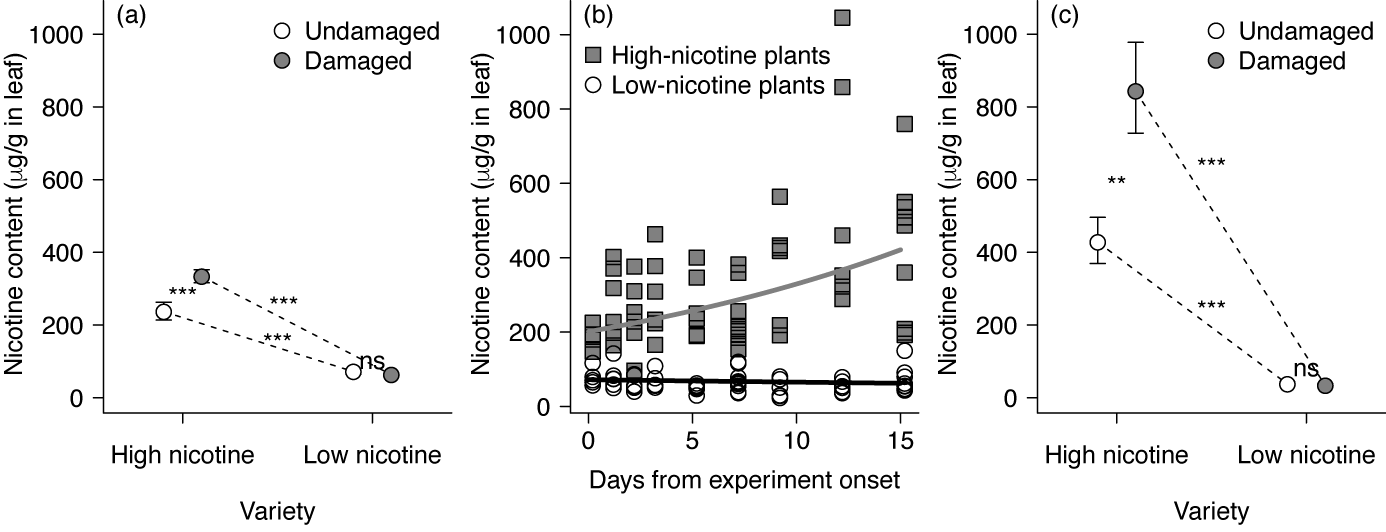


**Fig S1.** Nicotine content in *Nicotiana tabacum.* (a) Differences in the least-squares mean (± SE) nicotine content in leaves between leaf varieties and damage treatments. Nicotine content was adjusted for the effects of covariate factor (i.e., days from the experiment at onset). (b) Temporal variations in nicotine content. (c) Differences in least-squares mean (± SE) nicotine content in new-produced leaves between varieties and damage treatments.

# **Appendix S3. Volatile organic compounds**

To compare the levels and herbivore-induced release of volatile organic compounds (VOCs) emitted from plants between high-nicotine and low-nicotine varieties of *Nicotiana tabacum*, VOCs were measured on 2 June 2016 at the Center for Ecological Research (CER), Kyoto University in Otsu, Japan (34^o^58’ N, 135^o^57’ E). *Nicotiana* plants were grown in a greenhouse of CER, with herbivorous insects excluded using enclosures (see main text). From these plants, five individuals of each variety were selected and transported to a laboratory. We collected headspace VOCs from the plants using Twister (polydimethylsiloxane [PDMS] coating stir bar, film thickness 0.5 mm, 10 mm length, Gerstel GmbH & Co. KG). Headspace VOCs from each plant enclosed with 20-L polyethylene terephthalate bag were collected on two Twisters for 2 hrs. Then, we applied leaf damage to all plants. We punched 20-30 holes (1 cm in a diameter) on a given leaf at a middle positioned stalk to imitate foliar herbivory. The damaged plants were enclosed with 20-L plastic bag and we collected headspace volatiles as mentioned earlier. Thus, we collected VOCs twice from each plant, once before and once after leaf damage.

The collected volatile compounds were analyzed by GC-MS (GC: Agilent Technologies, Inc. 6890 with HP-5MS capillary column: 30 m long, 0.25 mm I.D. and 0.25 um film thickness; MS: Agilent Technologies, Inc. 5973 mass selective detector, 70 eV) equipped with a thermo desorption system (TDS), a cooled injection system (CIS), and a cold trap system (CTS) (Gerstel GmbH & Co. KG). Headspace volatiles collected on a Twister were released from the PDMS by heating in the TDS at 200°C for 4 min, within a He flow. The desorbed compounds were collected in the CIS at –130°C, and then the collected compounds were release from CIS by heating. The desorbed compounds were collected again in the CTS at –50°C, and then flash heating of the CTS provided sharp injection of the compounds into the capillary column of the gas chromatograph to which the CTS was connected. GC-oven temperature was programmed to rise from 40°C (9 min. hold) to 280°C at 5°C min^–1^. The compounds were identified by comparing their mass spectra to those of the database (Wiley7N).

We detected six VOCs emitted from the intact and/or damaged plants. We analyzed peak area of each VOC with generalized linear (mixed) models (GLM or GLMMs) carried out in SAS 9.4 (SAS Institute Inc.). The models involved gamma distribution (ln-link function) and included variety (high nicotine or low nicotine), treatment (intact or damaged), and their interactions as fixed factors and ln(wet weight of plants) as a covariate. Mixed models were necessary for the models, except for analysis of leaf alcohol (see below). The volatile collection was conducted twice as plants without and with leaf damages, hence we used GLMM with a model of compound symmetry to account for the correlated responses associated with this repeated measurement.

The composition of VOCs differed slightly between varieties (Table S2). Leaf alcohol (IUPAC nomenclature of chemistry; (*Z*)-3-hexen-1-ol) was only found in damaged plants without differences between varieties, indicating that emission of the leaf alcohol is damage-induced response. High-nicotine plants had 20% greater nicotine ((*S*)-pyridine 3-(1-methyl-2-pyrrolidinyl)) than low-nicotine plants, whereas low nicotine plants had 10% less beta caryophyllene (terpenes, (*E*)-beta-caryophyllene) than high nicotine plants (Fig. S2). We also detected norsolanadione ((3*E*) 3-nonene-2,8-dione) and two other terpenes (caryophyllene oxide and an indefinite terpene [tricyclene or cyclofenchene]) and these compounds did not differ between varieties or treatments (Table S2). Plant size did not influence VOCs in either case.

| Table S2. Results of generalized linear (mixed) models of the effects of variety (high nicotine or low nicotine) and foliar damage (intact or damaged) on the levels of volatile organic compounds in *Nicotiana tabacum*. | | | | | | |
| --- | --- | --- | --- | --- | --- | --- |
|  | Leaf alcohol^#1^ | Nicotine | Norsolanadione | Terpene^#2^ | Terpene^#3^ | Terpene^#4^ |
| Variety (high nicotine or low nicotine) | *F*_1,7_ = 2.51 | *F*_1,6.50_ = 24.60** | *F*_1,5.76_ = 3.92 | *F*_1,7.06_ = 13.78** | *F*_1,6.72_ = 5.34 | *F*_1,6.54_ = 1.63 |
| Leaf damage (damaged or undamaged) | – | *F*_1,7.72_ = 0.39 | *F*_1,8.10_ = 2.80 | *F*_1,7.50_ = 0.27 | *F*_1,7.99_ = 1.36 | *F*_1,7.88_ = 0.63 |
| Variety x leaf damage | – | *F*_1,7.72_ = 1.07 | *F*_1,8.10_ = 0.24 | *F*_1,7.50_ = 0.85 | *F*_1,7.99_ = 2.29 | *F*_1,7.88_ = 0.02 |
| Ln(wet mass per plant) | *F*_1,7_ = 1.95 | *F*_1,6.36_ = 0.09 | *F*_1,5.94_ = 0.01 | *F*_1,6.97_ = 1.70 | *F*_1,6.57_ = 2.25 | *F*_1,6.40_ = 0.04 |
| ** *P* < 0.01. | | | | | | |
| ^#1^ This compound was only found in damaged plants. | | | | | | |
| ^#2^ *β*−Caryophyllene | | | | | | |
| ^#3^ Caryophyllene oxide | | | | | | |
| ^#4^ Tricyclene or Cyclofenchene | | | | | | |


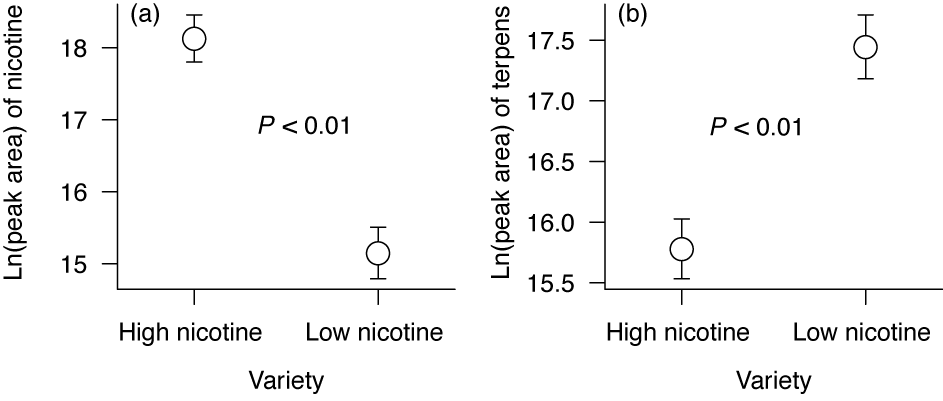


**Fig. S2.** The level of volatile organic compounds in *Nicotiana tabacum.* (a) Differences in the least-squares mean (± SE) peak area of nicotine (a) and *β*-caryophyllene (terpenes, b) between varieties. Peaks of VOCs were adjusted for the effects of leaf damage treatments and plant size.

# **Appendix S4. Grasshoppers' choice of plants**

To examine the effects of volatile organic compounds (VOCs) as attractants or repellents to grasshoppers, we studied feeding preference of the grasshopper, *Atractomorpha lata*, in the laboratory. Grasshoppers are common herbivores of *Nicotiana tabacum* in our common field, Center for Ecological Research (CER), Kyoto University in Otsu, Japan. We used a choice bioassay with two *Nicotiana* varieties (Burley 21, hereafter high-nicotine plants; LA burley 21, hereafter low-nicotine plants), selected for their different nicotine levels. We conducted two bioassay experiments using *Nicotiana* plants grown a common garden of CER. Plant leaves and grasshoppers for the experiments were collected from mature plants just before the experiment. Each experiment was conducted in a transparent acrylic plastic cage (60 cm x 60 cm x 60 cm) in a climate control room (25 ± 1 ^o^C, 60 ± 3 % RH, 16/8 hrs [day/night]). In each experiment, the grasshoppers (68 in total) were offered the choice of feeding on *Nicotiana* leaves set at both ends within the cage. We collected the leaves remaining 24 hours later, and measured remaining and damaged leaf area. Remaining leaves were oven-dried at 70 ^o^C for 48 hrs and weighed. The LMA (leaf mass: leaf area ratio) was calculated and leaf mass damaged was estimated as the products of LMA and leaf area remaining. All analyses were conducted with SAS 9.4 (SAS Institute Inc.) and considered experimental pair as a random factor to account for the correlated responses associated with this repeated measurement.

First, we assayed the levels of leaf herbivory for high-nicotine and low-nicotine plants to examine the grasshoppers' preference for feeding on leaves between varieties for 5 days during 18-25 August 2016. The high-nicotine and low-nicotine plants’ leaves were set at each end within the cage and this choice-experiment was conducted 24 times. Mass of damaged leaves did not differ between varieties (generalized linear mixed model [GLMM] with gamma distribution [log link], considering variety as a fixed factor: *F*_1,12.83_ = 0.19, *P* = 0.67), indicating that grasshoppers showed no feeding preference to a particular variety with different levels of volatile nicotine.

Second, we assayed the levels of leaf herbivory for low-nicotine plants near low-nicotine or high-nicotine plants to examine the effects of neighboring plants on the feeding preference of grasshoppers for 3 days during 6-8 August 2016 (in total 12 experiments). Leaves of low-nicotine plants were set at both ends within a cage, and additional leaves of high-nicotine or low-nicotine plants enclosed by a meshed nylon bag were set beside of leaves of low-nicotine LA plants. Air flow occurred over the leaves in mesh bags, but these leaves were not exposed to grasshopper attacks. Thus, we evaluated grasshopper behavior by allowing them to choose leaves of low-nicotine plants with and without the smell of neighboring high-nicotine plants. The extent of mass of damaged low-nicotine plant’s leaves was not significantly different between plants with and without neighboring high-nicotine plants (GLMM with gamma distribution [log link], considering absence/existence of neighboring high-nicotine plants as a fixed factor: *F*_1,7.42_ = 1.09, *P* = 0.33). These results indicate that grasshoppers showed no feeding preference to low-nicotine plants’ leaves near low-nicotine versus high-nicotine plants.

These bioassay experiments demonstrate that grasshoppers show no feeding preferences to low-nicotine or high-nicotine plants. These two varieties involve differences in nicotine production and volatile organic compounds, but both contribute little to grasshopper feeding preference at least during the experimental period.

# **Appendix S5. Seasonal variation in flower production and insect visits**

Observations of flower production of *Nicotine tabacum* and insect visitors were conducted at 2-3 days interval throughout the observation period. Part of this data was also used during main experiment in a main text (data after 18 Aug 2015, day of year [doy] = 230). Data after 230 doy were eliminated from analyses because plant conditions (e.g., longevity of individuals) were so heterogeneous. Here, we show seasonal variation in total number of flowers open and insects observed within the plot (12 m x 12 m) on each observation day (Fig. S3). In statistical analyses, we divided plot-census data in three phases (i.e., pre-flowering, flowering, and fruiting), considering overall conditions of plant-growth stage and emergence phenology of plant visitors. Flowering showed a unimodal pattern: flowering started on 171 doy and peaked on 200 doy (Fig. S3). Leaf caterpillars on plants peaked on 173 doy during the pre-flowering phase. Grasshoppers increased with time, though the pattern differed between varieties. Specifically, the increase in grasshopper numbers accelerated on high-nicotine plants and decelerated on low-nicotine plants, resulting in larger difference between them as time passed. Seed predators occurred in fruiting phase, and they decreased with time as newly-produced fruits decreased.


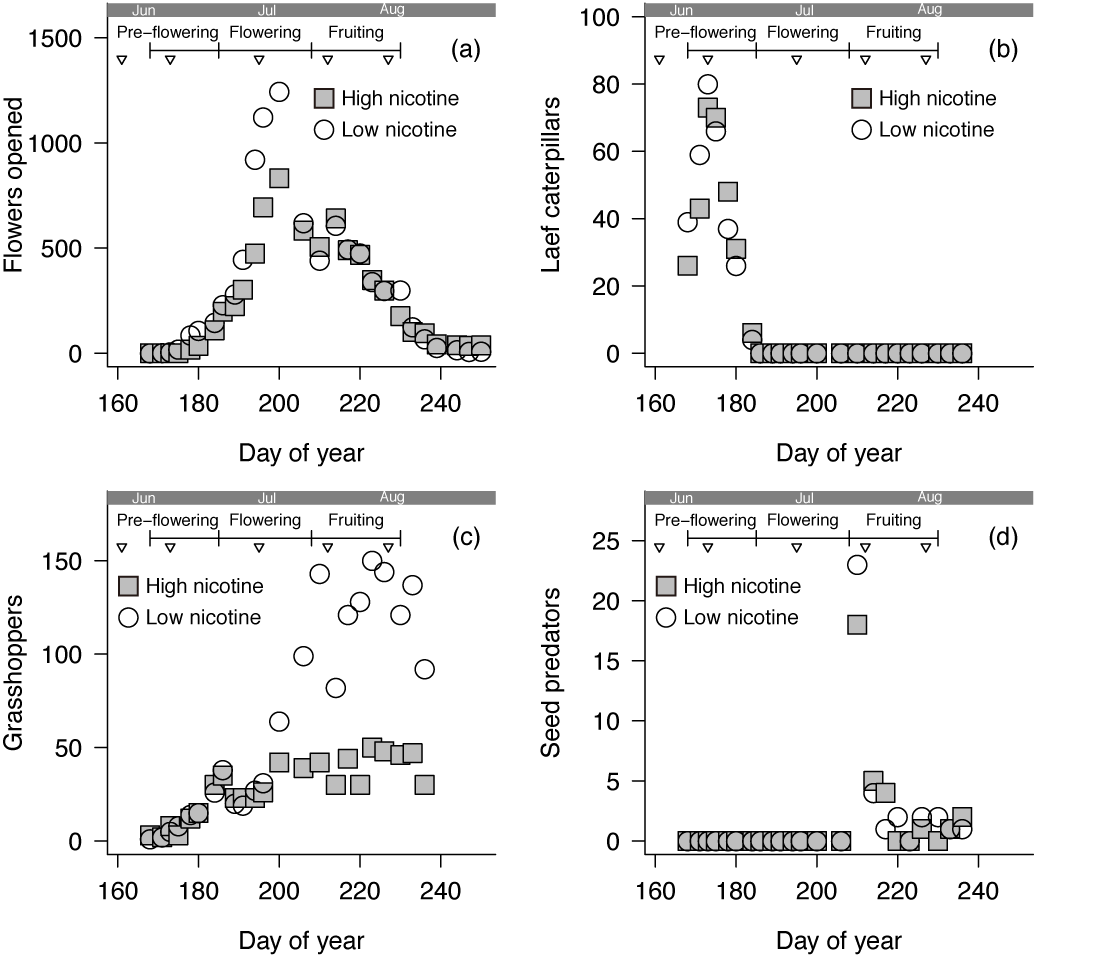


**Fig. S3.** Flowering patterns of *Nicotiana tabacum* (squares; high-nicotine plants, circles; low-nicotine plants) and seasonal variations in insects on the plants: (a) number of flower open, (b) number of leaf caterpillars on plants, (c) number of grasshoppers on plants, and (d) number of seed predators. Downward triangles indicate date of counting numbers of leaves and leaf herbivores (see main text).
